# Model‐Based Network Meta‐Analysis: A Framework for Evidence Synthesis of Clinical Trial Data

**DOI:** 10.1002/psp4.12091

**Published:** 2016-08-01

**Authors:** D Mawdsley, M Bennetts, S Dias, M Boucher, NJ Welton

**Affiliations:** ^1^ School of Social and Community MedicineUniversity of BristolBristolUnited Kingdom; ^2^ Pharmacometrics Group, Pfizer Ltd.SandwichKentUnited Kingdom

## Abstract

Model‐based meta‐analysis (MBMA) is increasingly used in drug development to inform decision‐making and future trial designs, through the use of complex dose and/or time course models. Network meta‐analysis (NMA) is increasingly being used by reimbursement agencies to estimate a set of coherent relative treatment effects for multiple treatments that respect the randomization within the trials. However, NMAs typically either consider different doses completely independently or lump them together, with few examples of models for dose. We propose a framework, model‐based network meta‐analysis (MBNMA), that combines both approaches, that respects randomization, and allows estimation and prediction for multiple agents and a range of doses, using plausible physiological dose‐response models. We illustrate our approach with an example comparing the efficacies of triptans for migraine relief. This uses a binary endpoint, although we note that the model can be easily modified for other outcome types.

**Study Highlights**

**WHAT IS THE CURRENT KNOWLEDGE ON THE TOPIC?**

☑ Methods to assess consistency in MBMA are limited. NMA methodology gives a self‐consistent set of effect size estimates, but limited work has been done to incorporate dose response.

**WHAT QUESTION DID THIS STUDY ADDRESS?**

☑ To allow dose response modeling within an NMA framework.

**WHAT THIS STUDY ADDS TO OUR KNOWLEDGE**

☑ The methods presented here allow arbitrary dose‐response models to be incorporated into an NMA, allowing prediction of compound efficacies across the studied dose range.

**HOW THIS MIGHT CHANGE DRUG DISCOVERY, DEVELOPMENT, AND/OR THERAPEUTICS**

☑ By making full use of trial data and pharmacologically derived dose response models, these techniques have the potential to accelerate the drug development process, while being assured of the consistency of evidence being used in the evidence synthesis.

Model‐based meta‐analysis (MBMA) has become an increasingly important tool in drug development to inform study design and decision‐making since it was first proposed (by Mandema *et al*.^1^). By including a dose and/or time response model in the meta‐analysis it is possible to compare treatments at doses and/or times that have not been directly compared in head‐to‐head trials, potentially reducing the number of trials required, allowing the competitive landscape to be surveyed, and reducing the risk of late stage trial failure.^2^ The dose‐response and time course patterns have been modeled using a variety of functional forms, including maximum effect (Emax)^1^ and linear^3^ models.

Network meta‐analysis (NMA)^4^, ^5^ provides a method to combine evidence on relative effects from comparative randomized controlled trials (RCTs) that form a connected network (where a path can be drawn between any two treatments). NMA is increasingly being used by reimbursement agencies,^6^, ^7^ where the focus is on making decisions about relative efficacy and cost‐effectiveness based on late stage and post filing RCT evidence. In contrast, MBMA tends to be used throughout the drug development process. NMA combines all evidence simultaneously, which means that both direct and indirect evidence contribute to relative effect estimates. When there are “loops” in the network then there is both direct and indirect evidence on the relative effects relating to that loop. For example, in **Figure** 1 there is direct evidence comparing sumatriptan with eletriptan but also indirect evidence from the sumatriptan vs. placebo trials and the eletriptan vs. placebo trials. Where both direct and indirect evidence exist, NMA provides a means of assessing the agreement between both types of evidence.^8^ Although model estimates of treatment effects will be consistent, the underlying data may not be. Where there are differences between direct and indirect evidence this is termed “inconsistency.”

**Figure 1:**
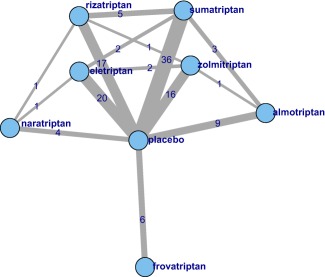
Network of treatments in our network meta‐analysis (NMA). Each treatment is represented by a node. Where direct trial evidence exists, treatments are joined by a line, the width of which is proportional to $\sqrt{\text{number}\;\text{of}\;\text{comparisons}}$. The figures on each edge indicate the number of treatment arms for each comparison.

**Figure 2:**
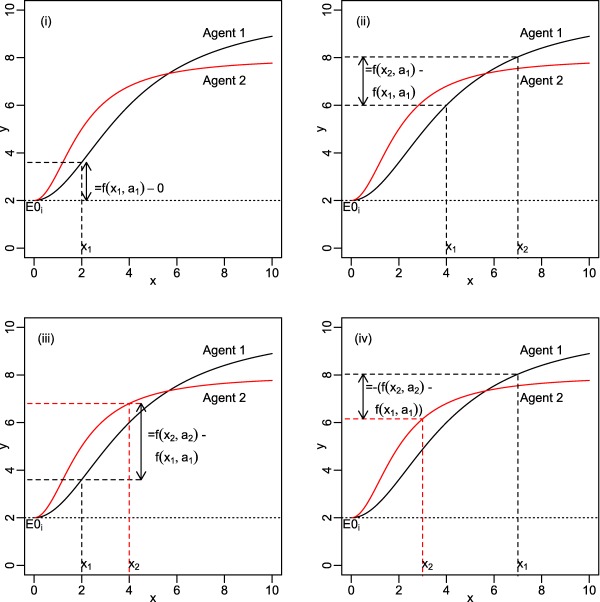
Schematic diagram illustrating how Eq. 7 picks out the correct relative effect for each comparison. In sub‐figure ***i***, dose *x_i_* of agent 1 is compared to placebo. In ***ii***, two different doses of agent 1 are compared. In ***iii*** and ***iv***, different doses of agents 1 and 2 are compared.

**Figure 3:**
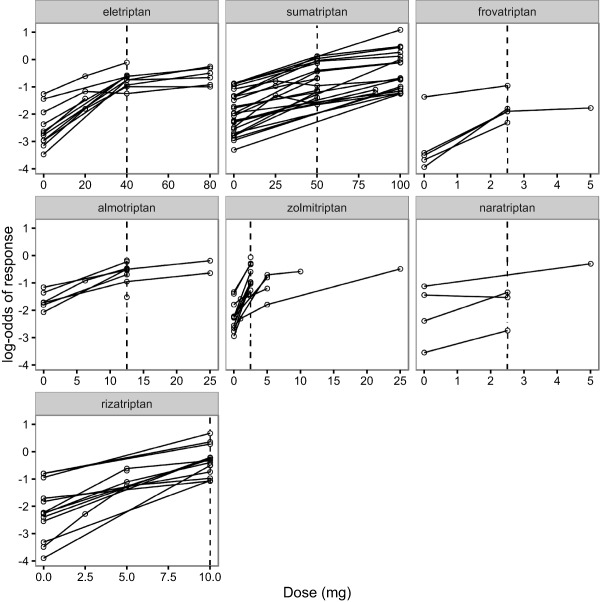
Plots of the log‐odds of patients with headache‐free response at 2 hours for each treatment as a function of dose. Each drug's common dose is shown by a vertical dashed line.

**Figure 4:**
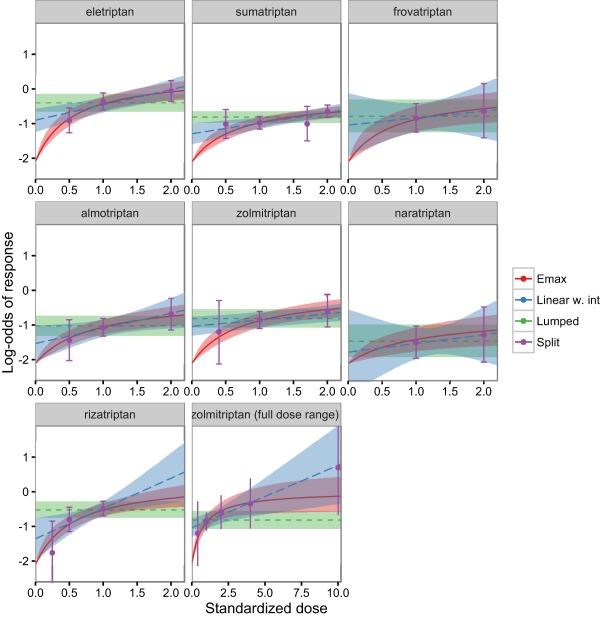
Model predictions from the lumped and split network meta‐analyses (NMAs) and the maximum effect (Emax) model‐based network meta‐analysis (MBNMA). The lumped results cannot be ascribed to any particular dose, so we show them across the whole dose range. Medians and 95% credible intervals are shown. The mean from a random effects model for placebo response across all placebo controlled trials was used to produce predictions for a typical trial.

In contrast to MBMA, the synthesis of multiple doses of the same agent is more limited within an NMA. Typically, different dose levels are either “lumped,” (i.e., assumed to have the same efficacy), or “split” where each agent by dose combination is considered as a separate treatment. Lumping increases the between‐study heterogeneity and the risk of inconsistency in the network. Interpretation of the results from a lumped NMA (a weighted average across all doses included for each agent) is also unsatisfactory. In contrast, a split analysis will typically have smaller between‐study heterogeneity, but will estimate effect sizes with lower precision, because each contrast is informed by fewer trials. A split network may even become disconnected, meaning that some treatments cannot be compared.

Del Giovane *et al*.^9^ have proposed models that treat different doses of the same agent as “similar” (exchangeable) using a hierarchical model with dose within agent, and by modeling adjacent doses with a random walk process, but the interpretation of estimates from these models can be difficult. Owen *et al*.^10^ proposed a hierarchical model that assumes a monotonic but nonparametric dose response between nodes representing different doses of the same drug, however, this does not allow prediction of effects at doses not included in the RCTs. Thorlund *et al*.^11^ assumed a linear dose response model on the log‐odds scale in an NMA for doses at half or double each drug's “common” dose. This approach allows for prediction at doses not included in the RCTs, but a linear model may be too restrictive and is not biologically plausible in many applications.

We propose a new framework, model‐based network meta‐analysis (MBNMA), that combines the advantages of MBMA and NMA. We illustrate our approach with an example that uses a binary endpoint to compare the relative efficacies of triptans for migraine relief. We also describe how the Bayesian framework proposed can be used to model other output types, such as continuous outcomes.

The study is organized as follows. We begin by setting out NMA and MBMA models before describing our MBNMA framework. We then describe our illustrative example and present results. We end with a discussion contrasting the different methods and outline challenges for future work.

## MATERIALS AND METHODS

### Description of models

#### Model‐based meta‐analysis

MBMA has typically been used in pharmaceutical decision‐making, whether it be to inform study design, define decision criteria for a successful study with a new compound, or simply learning about different attributes of competitor drugs. For much of drug development, the emphasis is on learning rather than confirming,^12^ leading to the focus being less on a single end of study endpoint for an approved dose and more on dose response and time‐course as well as relevant covariates. In the future, however, it is likely that MBMA will play an increasing role in regulatory submissions and reimbursement decisions.

It is possible to synthesize different dose and/or time points within a single meta‐analysis, by modeling plausible dose or time responses.^1^ The Emax model is widely used to model the effect of a drug as a function of dose, *x*, and takes the following form:

(1) $$\text{Response}=E0_{i}+\frac{{\text{Emax}50}_{t_{i,k}}+x_{i,k}}{\text{ED}50_{t_{i,k}}+x_{i,k}}$$

The model assumes there is an Emax relative to placebo response (*E*0) corresponding, physiologically, to the drug saturating the body's receptors.^13^ The ED50 parameter represents the dose at which half the maximum effect is reached.

Because RCTs are designed to provide estimates of relative effects, not absolute effects, the estimated outcome for *E*0_*i*_ of Eq. 1 is treated as a “nuisance” parameter. For each of the other arms, a relative effect is estimated for the treatment on that arm relative to the control arm treatment, thereby ensuring that the principle of concurrent control is respected. From a learning perspective there is often a desire to assess covariates that may influence the size of the placebo response. This might be with the aim of designing a study with a population in which placebo response tends to be lower. There tend to be two approaches to fitting *E*0_*i*_. One is to assume the placebo response is different for each study (modeling heterogeneity as independent, the nuisance parameter approach) while the other is to fit with random effects (hence, modeling heterogeneity as random but similar across studies). In this latter approach, the principle of concurrent control is violated, which can lead to biased estimates of relative effect.^14^ The extent to which this will be a problem will depend on the variation in sample size ratios between groups within trials, across the studies.^15^ Although this has been discussed from a linear model point of view, the authors are not aware of any such research into the significance of this issue in nonlinear MBMA models. Often the placebo effect is of interest in itself, making it attractive to put a random effects model on the placebo arm. This is better achieved by a separate analysis of the placebo arms, which delivers estimates of placebo effect, but avoids introducing bias in the estimation of the relative effects.

Time‐course of response is often fitted using an Emax model or a simpler exponential model. It is not unusual to model dose and time with a single MBMA.^16^, ^17^

#### Network meta‐analysis

NMA was first introduced by Higgins and Whitehead^4^ and was further developed by Lu and Ades^18^ and Dias *et al*.^19^, ^20^ In order to respect the principle of concurrent control, a relative effect relative to the control arm treatment effect (which is treated as a nuisance parameter) is calculated. The general model is:

(2) $$g(\theta_{i,k})=\{\begin{array}{cc} \mu_{i} & \text{when}\;k=1\\ \mu_{i}+\delta_{i,k} & \text{when}\;k\geq 2 \end{array}$$

where *g* is a link function that transforms the outcome onto an appropriate scale (e.g., the logistic function for the log‐odds scale for binary outcomes, or the identity function for continuous outcomes),^19^ *θ* is a parameter that the data provide information on directly through the likelihood (e.g., mean or proportion), *μ_i_* is the effect on arm 1 of study *i*, which we treat as a nuisance parameter, and *δ_i_*_,__*k*_ is the relative effect for arm *k* of study *i*, relative to the reference treatment of the study. Either a fixed effect model, or, if there is heterogeneity in relative effect estimates between studies, a random effects model can be fitted. For a fixed effect NMA model all the studies are assumed to be estimating the same underlying effect for the treatments that they compare, so we set:

(3) $$\delta_{i,k}=d_{t_{i,1},t_{i,k}}$$

where *t_i_*_,1_ and *t_i_*_,__*k*_ are the treatments in the first and *k*th arms of the *i*th trial and *d_b_*_,__*k*_ is the relative effect of treatment *k* relative to treatment *b*. The fixed‐effect model extends naturally to a random‐effects model, where we have:

(4) $$\delta_{i,k}∼N(d_{t_{i,1},t_{i,k}},\sigma^{2})$$

so that each study is estimating a true underlying effect, and these are assumed to be exchangeable. The *σ* 2 represents the between study variability in relative effects, which is usually assumed to be common to all comparisons. The random effects generated by multi‐arm trials are correlated; this must be accounted for in the model, as described in Dias *et al*.^19^

An NMA consisting of *s* treatments can be defined in terms of *s*−1 basic parameters, *d*_1,2_,*d*_1,3_,…*d*_1,__*s*__−1_, which estimate the effects of all treatments relative to the reference treatment. All other treatment contrasts are defined as functions of the basic parameters, ensuring consistency of all the estimated contrasts.^19^ This follows from the assumption of exchangibility^5^:

(5) $$\begin{array}{c} d_{c,k}=d_{1,k}-d_{1,c} \end{array}$$

#### Model‐based network meta‐analysis

We extend the MBMA and NMA frameworks to an MBNMA as follows. Following the NMA framework, we specify an appropriate likelihood and scale for our data and model. In our illustrative example, we have a binary outcome, so we use a binomial likelihood and logistic link function, and interpret our relative effects, *d*, as log‐odds ratios.

(6) $$\text{logit}(p_{ik})=\{\begin{array}{cc} \mu_{i} & \text{when}\;k=1\\ \mu_{i}+\delta_{i,k} & \text{when}\;k\geq 2 \end{array}$$

As with the NMA approach, other likelihoods and link functions, appropriate to the outcome scale (e.g., continuous outcomes using a normal likelihood, and natural link), can be used (see Eq. 2 and Dias *et al*.^20^).

We treat the log‐odds of an event in arm one of each trial as a nuisance parameter. In a fixed effect model, the relative effect for arm *k* relative to arm 1 in study *i* is modeled as:

(7) $$\delta_{i,k}=f(x_{ik},t_{ik})-f(x_{i1},t_{i1})$$

where *t_ik_* and *x_ik_* are the agent index and dose, respectively.

The *f*(*x_ik_*, *t_ik_*) represents the functional form of the dose‐response relationship (assumed common to all agents). Eq. 7 ensures that the correct comparison is made between the agent and dose on arm k with the agent and dose on arm one, as illustrated in **Figure** 2.

For a random effects model, we assume that there is between‐study variability around the estimated relative effects after accounting for the agents and doses that have been compared via the dose‐response model, given by:

(8) $$\delta_{i,k}∼N(f(x_{ik},t_{ik})-f(x_{i1},t_{i1}),\sigma^{2})$$

Multi‐arm trials are dealt with in the same way as a standard NMA.^19^

In principle, any function of dose and treatment could be used. We consider the linear model:

(9) $$f(x_{ik},t_{ik})=b_{t_{ik}}+d_{t_{ik}}x_{ik}$$

and a linear model excluding the intercept term, $b_{t_{ik}}$.

For the Emax model we take:

(10) $$f(x_{ik},t_{ik})=\frac{{\text{Emax}}_{t_{ik}}x_{ik}}{{\text{ED}50}_{t_{i,k}}+x_{ik}}$$

Because ED50 is a dose, it is necessarily positive, so we model this parameter on the log‐scale. We do not make such an assumption for Emax to allow the possibility that treatments may have either beneficial or harmful effects. We note that the Emax model will require a rich data structure to estimate all the parameters, and, in many practical cases, may not be fully identifiable. We therefore consider models that assume that one or both of Emax and ED50 are exchangeable across agents within drug class, ${\text{Emax}}_{t}∼N(\mu_{\text{Emax}},\sigma_{\text{Emax}}^{2})$, and/or ${\text{ED}50}_{t}∼N(\mu_{\text{ED}50},\sigma_{\text{ED}50}^{2})$. This allows the model to “borrow strength” across agents in order to improve estimation.^21^, ^22^, ^23^

### Estimation

MBMAs are typically estimated using frequentist methods in nonlinear modeling software, such as NONMEM,^24^ R^25^, and S‐Plus,^26^ although they could also be specified using Bayesian methods.

NMA models have been estimated using frequentist and Bayesian approaches. We follow Dias *et al*.^19^ and adopt a Bayesian approach. We fit our models using JAGS 3.4.0,^27^ which has a very similar modeling language to WinBUGS (code included in the **Supplementary Material**). The language's flexibility allows us to extend the NMA framework to MBNMA. Vague normal prior distributions (N(0,1000)) are given to the basic parameters *d*_1,__*k*_, nuisance parameters *μ_i_*. We model the between‐study SD and the SDs on the exchangeable parameter distribution using a uniform(0, 4) prior. As meta‐analysis models may be sensitive to the choice of prior for this parameter, the analysis was repeated for the Emax MBNMA model using a half‐normal prior with precision 100, with almost identical results.

The parameter estimates from the models can be used to make predictions for response for dose by agent combinations. This is achieved by applying the estimated relative effect for each treatment to an estimate of the placebo effect. We obtain the placebo effect estimate using a random effects model for the placebo arms in the trials that included placebo, calculated separately to the MBNMA. We use the mean of the random effects distribution to simulate predictions for a “typical” trial. An alternative approach is to draw simulations from the whole distribution to capture the uncertainty in this estimate together with uncertainty in the relative effect estimates to form prediction intervals.

### Model checking

Goodness of fit of MBMA is typically assessed using standard frequentist methods, such as likelihood ratio tests. Model fit of Bayesian NMA and MBNMA can be assessed by inspecting the posterior mean of the residual deviance,^28^ where lower values indicate better model fit. Models can also be compared using the Deviance Information Criterion (DIC),^29^, ^30^ which represents a compromise between model fit and complexity; smaller values are preferred, with differences of three or more considered meaningful. With nonlinear models it is important to use the “plug‐in” method for the effective numbers of parameters used to calculate DIC, as JAGS and WinBUGS default values can be unreliable for these models.^28^

Methods for assessing the consistency of evidence included in an MBMA are less well developed than in NMA. In NMA, the consistency of direct and indirect evidence in the whole network can be assessed by comparing the fit of the NMA model to an unrelated mean effect (UME) model (also known as an inconsistency model).^31^ The UME model estimates each treatment contrast using only direct evidence, while still assuming a common between‐study variance parameter. This approach makes no use of indirect evidence, and is equivalent to simultaneously performing separate pairwise meta‐analyses, assuming a common between‐study variance. The overall model fit and between‐study heterogeneity are compared to the full NMA model. The mean contribution of each data point to the posterior mean residual deviance can also be compared; when some points have substantially larger deviance contributions under the NMA model than the UME model this may be indicative of inconsistency.

This approach can be extended to an MBNMA. We compare the mean contributions to posterior mean residual deviance from each data point in the MBNMA models to a UME model, which relaxes the assumption of consistency and which assumes no functional relationship between dose and response (i.e., in which the treatments are equivalent to those in the split NMA).

### Illustrative example

We illustrate our model using published clinical trial data for the efficacy of triptans in migraine pain relief.^11^ The headache free at two hours' endpoint was used to compare the efficacy of the agents as a function of dose. We used data from patients who had a least one migraine attack, who were not lost to follow‐up, and who did not violate the trial protocol.^11^ Where this information was not available, the data were augmented with modified intention to treat data (i.e., excluding patients who, although randomized, did not suffer at least one migraine attack).

Our data‐set consists of 70 RCTs, comparing 7 triptans and placebo. **Figure** 1 shows the network of comparisons in the data, ignoring the doses of each comparison. **Figure** 3 shows plots of the log‐odds of response as a function of dose for each agent. A nonlinear dose response can be seen in some agents, however, for some agents the limited amount of data makes it difficult to infer an appropriate dose response model.

The trials contained dose information, which was standardized to multiples of each agent's “common” dose; the single dose indicated by the US Food and Drug Administration (with the exception of sumatriptan, where, following Thorlund *et al*.,^11^ a common dose of 50 mg was used).

We performed “lumped” and “split” NMAs using the code from the NICE TSD series.^20^ In the lumped model, we treated all doses for each drug as equivalent. When trials contained multiple arms for the same drug, we assumed that the relative effect for these arms was zero, noting that such trials would still contribute to our estimate of between study heterogeneity. In the split network, each combination of drug and standardized dose was treated as a separate treatment in our network of comparisons.

MBNMAs were fitted using (i) a linear dose response on the log‐odds scale (with and without an intercept term) and (ii) an Emax MBNMA, using the whole dose range available for each drug. As we had a limited dose range, the ED50 parameter in the Emax MBNMA proved difficult to estimate reliably. We therefore fitted models that assumed that either or both parameters were exchangeable about respective common means.

All models were run on three independent chains for 40,000 iterations following 40,000 burn‐in iterations, with a thinning parameter of 10. Gelman's $\hat{r}$ statistic^32^ and visual inspection of the chains were used to assess convergence.

## RESULTS

**Table** 1 shows goodness of fit statistics for the lumped and split NMAs, and for the MBNMAs described above, however, we were unable to identify the between agent SD for the “Emax (Emax exch.)” model, so results for this model must be interpreted with caution. **Table** 2 shows the estimate of the class means and SDs for the models with class effects.

**Table 1:** Model fit statistics for the NMA and MBNMAs considered in the main text

Model	DIC	pD	Residual deviance	Between‐study SD
Lumped NMA	330.51	141.47	189.04	0.373 (0.289–0.469)
Split NMA	325.21	135.63	189.58	0.27 (0.178–0.376)
Linear MBNMA	337.70	154.68	183.02	0.556 (0.46–0.672)
Linear MBNMA w. intercept	320.98	132.29	188.69	0.274 (0.192–0.371)
Emax MBNMA	327.70	136.89	190.81	0.285 (0.193–0.392)
Emax (ED50 exch.)	321.75	130.23	191.52	0.249 (0.159–0.35)
Emax (Emax exch.)	327.51	136.45	191.06	0.292 (0.188–0.418)
Emax (Emax & ED50 exch.)	318.70	126.77	191.92	0.242 (0.16–0.335)
UME	345.58	136.46	209.12	0.22 (0.098–0.34)

DIC, Deviance Information Criterion; Emax, maximum effect; ED50, dose at which half maximum effect obtained; MBNMA, model‐based network meta‐analysis; NMA, network meta‐analysis; pD, parameters; UME, unrelated mean effect.

The DIC and effective number of pDs are calculated using the plug‐in method. Mean residual deviance and median between‐study heterogeneity (with 95% credible intervals) are reported. The data contain 182 data points; we would expect each to contribute ≈ 1 to the residual deviance.

**Table 2:** Class means and SDs

	*μ*_Emax_	*σ*_Emax_	*μ*_ED50_	*σ*_ED50_
ED50 exch.			0.766 (0.331–4.725)	1.639 (1.022–17.7)
Emax exch.	1.653 (1.155–2.393)	6.942 (1.167–19.028)		
Emax and ED50 exch.	2.114 (1.446–2.838)	0.518 (0.051–1.412)	0.676 (0.315–1.552)	1.539 (1.028–4.916)

Emax, maximum effect; ED50, dose at which half maximum effect obtained.

Estimates of ED50 have been converted to the natural scale. Medians and 95% credible intervals are shown. Owing to difficulties estimating *σ_Emax_*, we used a uniform(0,20) prior for this parameter.

Comparing the lumped and split NMA, the split NMA has the smaller DIC, indicating that, taking model complexity and fit into consideration, it should be preferred over the lumped model. As we would expect, the lumped model, which treats the different doses (and hence responses) of each agent as equivalent, has a larger between‐study heterogeneity than the split model.

Of the two linear models considered, the model that included the intercept term fitted the data better; it had a lower DIC and smaller between‐study heterogeneity. All the Emax MBNMAs have a similar level of between‐study heterogeneity, which is slightly larger than in the split model. The Emax MBNMA that assumes a class effect on ED50 gave lower heterogeneity and lower DIC statistics than those that did not.

We see that, according to the DIC statistic, the Emax MBNMA, which assumes exchangeable Emax and ED50 parameters, is the best compromise between‐model fit and complexity and has the smallest between‐study heterogeneity. We therefore do not consider the other Emax MBNMAs in the remainder of this article.

We include a visual predictive check of this model in the **Supplementary Figure S1**, which suggests the model captures the data well. A plot of deviance contributions as a function of standardized dose (**Supplementary Figure S2**) shows that vast majority of deviance contributions are ∼1 (we would expect each data point to contribute ∼1 to the residual deviance), although there are some larger residuals for placebo response suggesting greater than expected baseline variability. The observed total residual deviance is 191.92; comparable to the number of data points (182).

**Figure** 4 shows predictions for a typical trial of the proportion of headache‐free patients as a function of dose for each agent under the lumped and split NMA and the linear (with intercept) and Emax MBNMAs. The Emax MBNMA tends to have a smaller prediction interval than the linear or split models, and appears to capture the agents' observed dose responses well.

The UME model gave a lower estimated between‐study SD than the MBNMA models, suggesting evidence of inconsistency. The results should therefore be interpreted cautiously and further exploration of inconsistency is required (e.g. using node‐splitting).

## DISCUSSION

We have shown how the NMA framework can be extended using ideas from the MBMA literature to incorporate dose‐response models. By applying the consistency equations from NMA at the level of the dose‐response curve we construct a model that ensures that the agents' dose‐responses are modeled in a coherent manner. In this framework, both direct and indirect evidence may inform our parameter estimates. The consistency of the direct and indirect evidence can be assessed with an unrelated mean effects model,^33^ although this does not make full use of the modeled dose‐response. We note that other approaches to assessing the consistency of direct and indirect evidence exist, such as node splitting,^8^ which assesses the consistency of each contrast where both direct and indirect evidence exists. Further work is required to extend the node‐splitting methodology to MBNMA.

We illustrated our method using a dichotomous end point (“headache‐free at 2 hours”) using a binomial likelihood. However, the methods are applicable to any generalized linear model with an appropriate likelihood and link function. Dias *et al*.^19^, ^20^ provide example WinBUGS codes for NMAs for many outcome types, such as using a normal likelihood to model a continuous endpoint. The methods presented here could be similarly adapted for other outcome types.

Many early phase studies report repeated measures over time. There have been applications of MBMA, which can simultaneously model dose and time course information.^1^, ^16^ In this article, we considered a single outcome at a single time point. We note that Jansen *et al*.^34^ modeled the time‐course of interventions for knee osteoarthritis using fractional polynomials,^35^ which allowed for between‐study heterogeneity on one of the fractional polynomial parameters. We plan to extend the MBNMA framework to simultaneously model dose and time course.

The MBNMA approach provides a more flexible modeling approach than either lumped or split NMAs. In a lumped NMA, we are unable to make predictions as a function of dose. Split NMAs do not assume any form of dose‐response relationship, and so can only be used to make predictions at trialed doses. This limits the utility of such models in a drug‐development context, where we may wish to make predictions at doses that have not yet been trialed for future studies. In a policy context, we are interested in licensed doses of agents, and so prediction for doses not included in RCTs is not an issue, however, including early phase studies with dose‐response information may improve precision of estimates of relative efficacy for doses that we are interested in, and may help to connect the network of evidence, allowing a wider range of agents to be compared. Furthermore, agents may not have been compared with an appropriate dose of a comparator agent and prediction of the relative efficacy of the agent under consideration against alternative doses of the comparator agent may be of interest. MBNMA models facilitate such comparisons.

The MBNMA approach we have outlined makes full use of all trial data, and models a dose response curve. The functional form chosen for the dose response could be informed by goodness of fit statistics (as in our example) and/or by pharmacological arguments. In this example, we found that the linear model with intercept fitted the data better than the model without an intercept, even though the intercept term is problematic to interpret from a pharmacological perspective; it can be thought of as representing nonlinearity as the dose falls to zero.

By modeling between‐study heterogeneity at the level of the adjusted outcome, we measure it on the same scale as the outcome, making its interpretation more straightforward. We had previously investigated models that allowed for between‐study heterogeneity in the Emax and/or ED50 parameters, but found they suffered from parameter identification issues, and create difficulties for multi‐arm trials.

In this example, where all drugs were triptans, we assumed that each of the model parameters was exchangeable about its own class‐specific mean in order to improve estimation and parameter identifiability. This extends naturally to the case where there are several classes of drugs being evaluated, where it might be appropriate to assume exchangeability within each drug class. In future work, we plan to conduct a simulation study to better understand the model's data requirements if we wish to avoid assuming exchangeability.

In summary, we have presented an MBNMA framework that combines evidence from RCTs comparing treatments (agent and dose combinations), respects randomization in the included RCTs, allows estimation and prediction of relative effects for multiple agents across a range of doses, uses plausible physiological dose‐response models, allows assessment of model fit and evidence consistency, and therefore has a valuable role in drug development and reimbursement decisions.

## Supporting information

Supporting Information

Supporting Information
